# Supplementary material for: Spin-momentum locking and spin-orbit torques in magnetic nano-heterojunctions composed of Weyl semimetal WTe2
Source: Nat Commun. 2018 Sep 28;9:3990. doi: 10.1038/s41467-018-06518-1 (PMC6162210; doi:10.1038/s41467-018-06518-1)
Supplement: Supplementary file 1 — Supplementary Information [file 41467_2018_6518_MOESM1_ESM.pdf]

Supplementary Materials for

Spin-momentum locking and spin-orbit torques in magnetic nano-heterojunctions composed of Weyl semimetal WTe_2

Peng Li,[1†] Weikang Wu,[2†] Yan Wen,[1†] Chenhui Zhang,[1] Junwei Zhang,[1] Senfu Zhang,[1] Zhiming Yu,[2] Shengyuan A. Yang,[2] A. Manchon,[1] andXi-xiang Zhang[1*]

[1]King Abdullah University of Science and Technology, Physical Science and Engineering Division, Thuwal 23955-6900, Saudi Arabia.

[2]Research Laboratory for Quantum Materials, Singapore University of Technology and Design, Singapore 487372, Singapore.

[†] These authors contributed equally to this work.

[*] Correspondence and requests for materials should be addressed to X.X.Z. (email: xixiang.zhang@kaust.edu.sa).

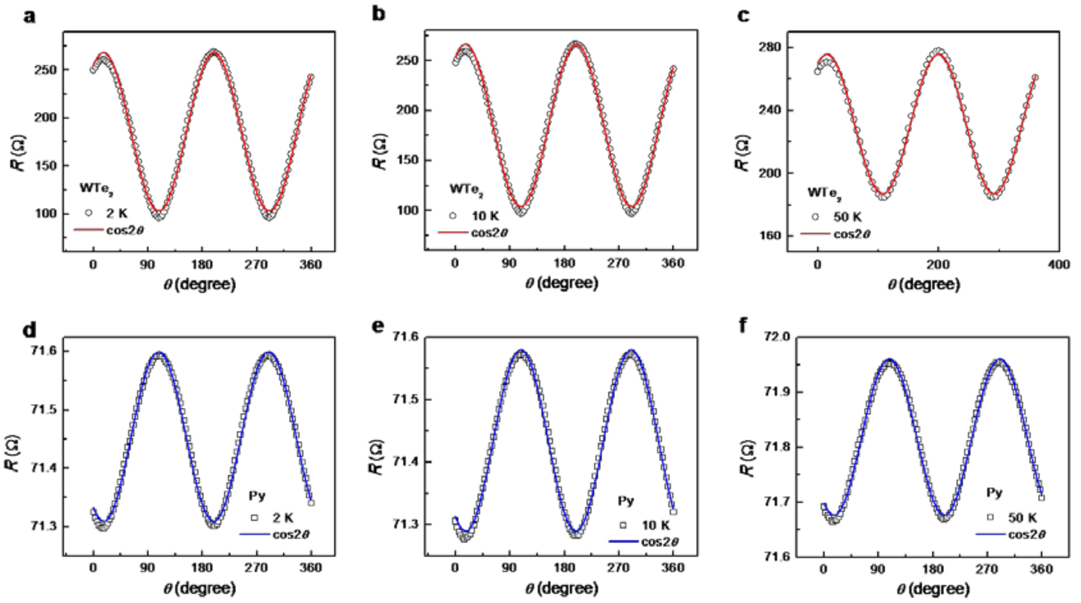

Supplementary Figure 1| Typical angular-dependent magnetoresistance of WTe_2 and Py.

The thicknesses of WTe_2 and Py are 20 nm and 6 nm, respectively. During the measurement of AMR, the current was along the *b* axis, and a magnetic field of approximately 9 T was rotated in the *ab* plane of the WTe_2. Both of the angular-dependent AMRs at different temperatures in WTe_2 (**a–c**) and Py (**d–f**) approximately follow $\cos 2\theta$, which is significantly different from the relation $|\cos\theta|$ in the WTe_2/Py bilayers, which measured T<100 K (Fig. 1d and Supplementary Fig. 3).

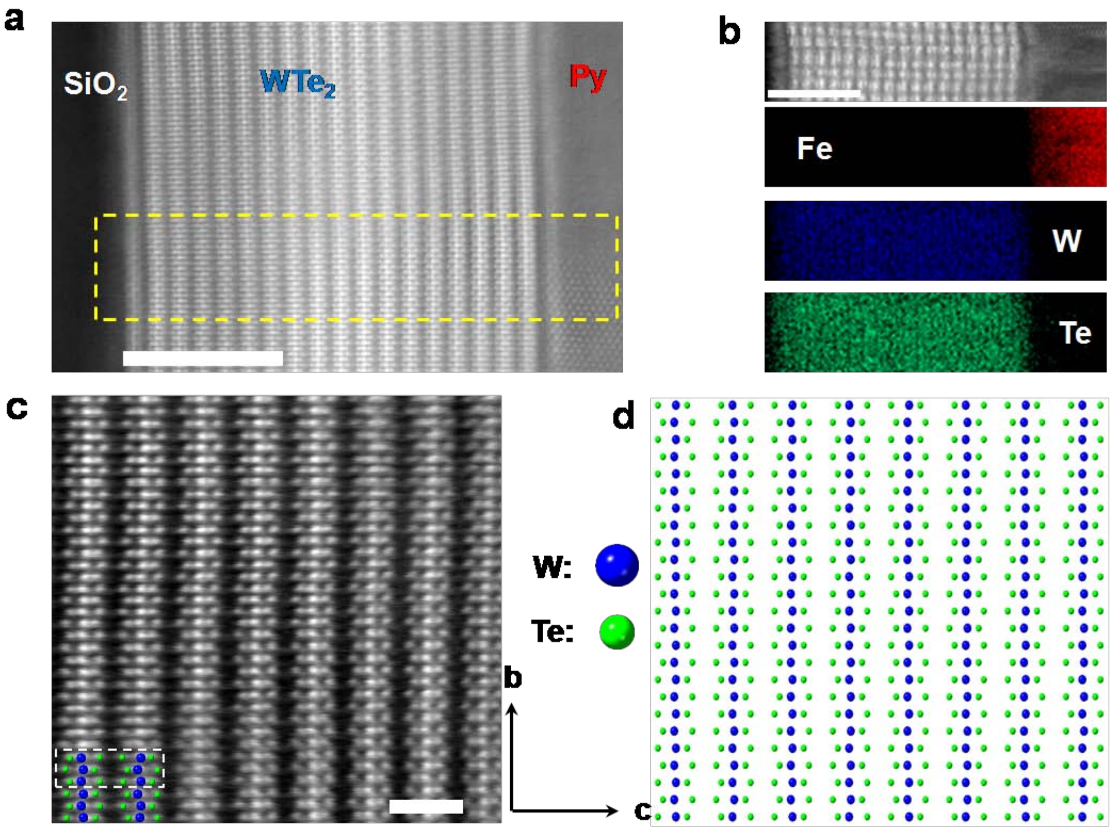

Supplementary Figure 2| High-angle annular dark-field image and electron energy loss spectroscopy mapping of WTe_2/Py bilayers.

a. Cross-sectional high-angle annular dark-field(HAADF) image of WTe_2/Py bilayers. The interface between WTe_2 and Py is very sharp and well-defined. This high-quality interface gives rise to the 2D electron transport behavior (Fig. 1d).

b. Electron energy loss spectroscopy (EELS) mapping of the WTe_2/Py interface (the dashed yellow rectangular region in **a**). The elements W, Te, and Fe are illustrated.

c. High-resolution HAADF image of WTe_2 (bc plane). The W (blue) and Te (green) atoms are added for clarity. The white dashed rectangle represents the unit cell of WTe_2.

d. The ideal crystal structure of WTe_2 in the bc plane is included for comparison.

The white scale bar is 5 nm in **a** and **b**, and is 1 nm in **c**.

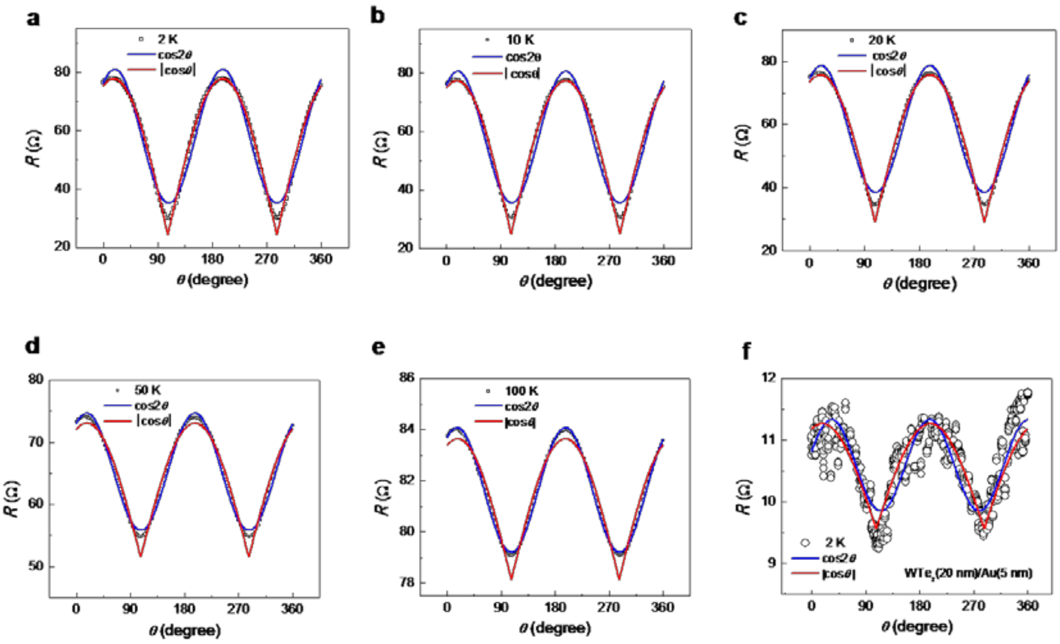

Supplementary Figure 3| Evolution of angular-dependent magnetoresistance in WTe_2/Py.

With an increase of temperature (**a–e**), 2D electron transport at the interface of WTe_2 and Py with atypical angular dependent of AMR relation $|\cos\theta|$ decays gradually and almost vanishes upon entering the range of 50–100 K. To confirm the 2D electron transport behavior, we also checked the AMR behavior in the WTe_2/Au bilayer (**f**). The typical feature of AMR $|\cos\theta|$ in WTe_2/Au with 2D electron transport behavior was also clearly observed at 2 K and 9 T. The emergence of 2D electron transport at the interface should be ascribed to the enhanced interface DOS (Supplementary Fig. 4).

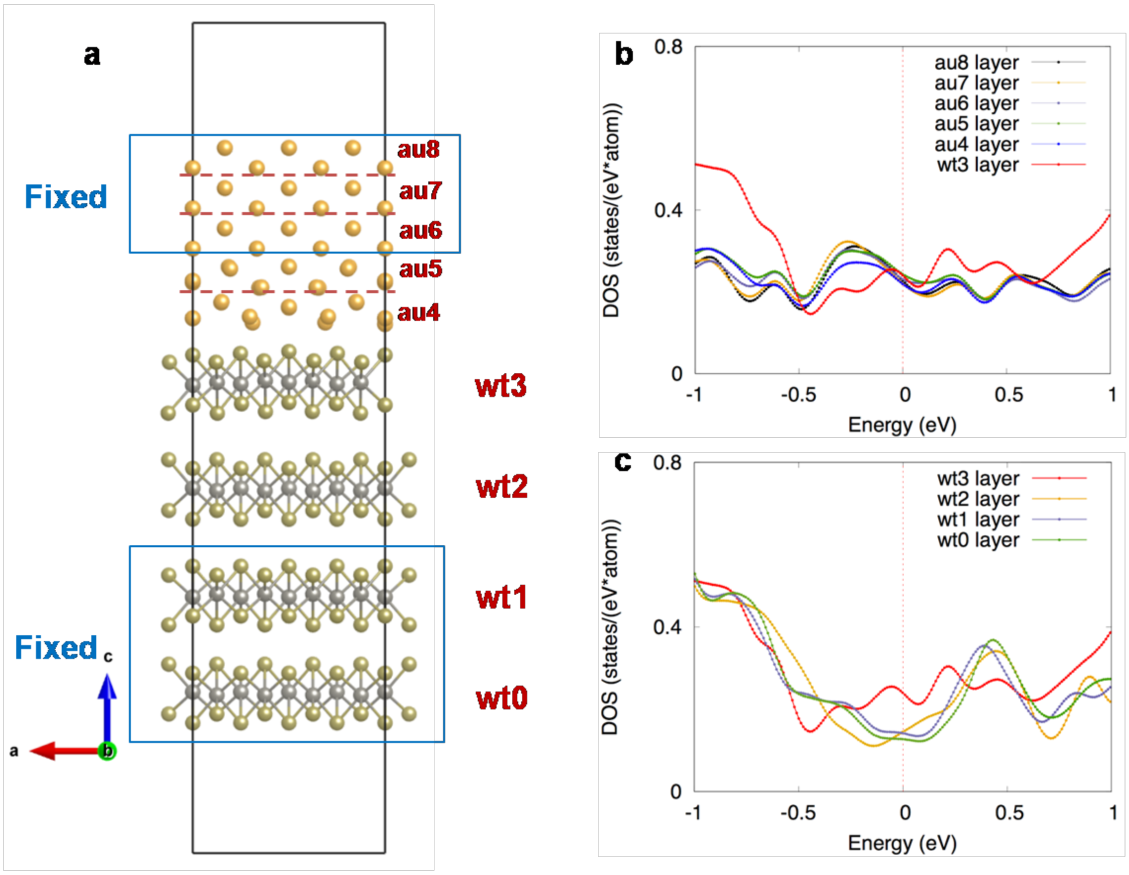

Supplementary Figure 4| WTe_2/Au interface density of states calculation.

a. A crystal model of the WTe_2/Au interface. The layers that are far from the interface, including the WTe_2 and Au layers, were fixed to perform the crystal optimization. A vacuum layer larger than 15 Å was used to eliminate the interaction between adjacent images.

b. The calculated DOS spectra of the separate Au layer near the Fermi level.

c. The calculated DOS spectra of the separate WTe_2 layer near the Fermi level. Obviously, the DOS of the WTe_2 layer nearest the interface was doubled enhanced as compared to the WTe_2 layer farthest from the interface.

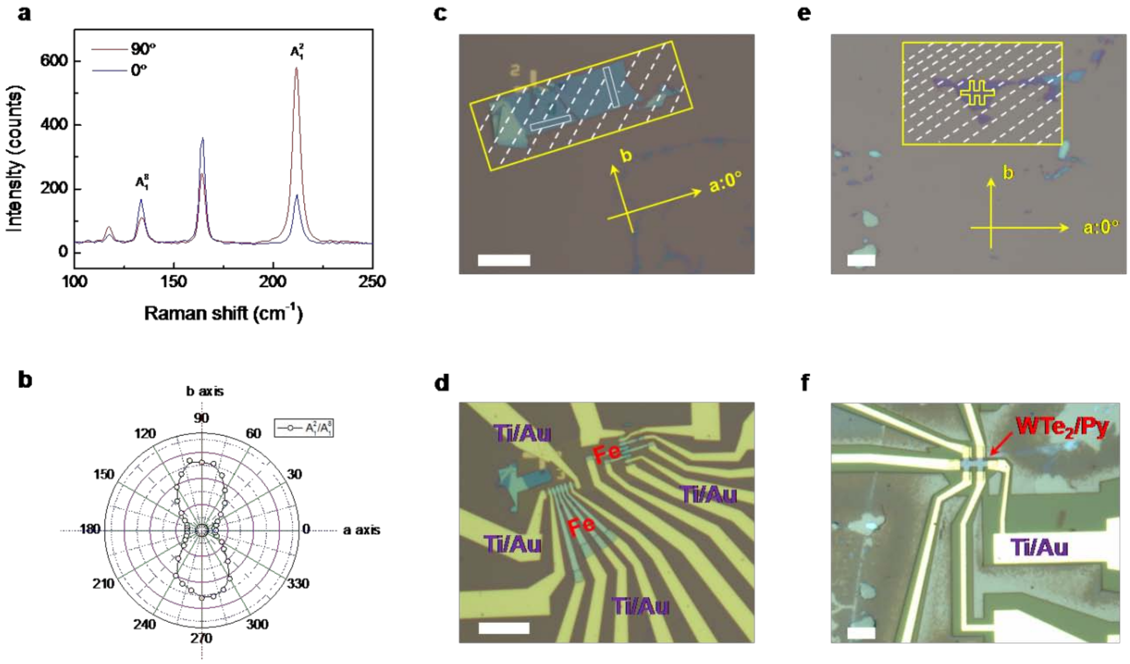

Supplementary Figure 5| Schematic of WTe_2 device fabrication with different crystal directions.

a. A typical polarized Raman spectrum of WTe_2 flakes along different angles. The intensity of the Raman spectra collected at θ = 0° and θ = 90° are significantly different, especially for the peaks A_1^8 (~130 cm^{-1}) and A_1^2 (~212 cm^{-1}).[1-3]

b. The extracted in-plane crystal-angle-dependent Raman intensities of A_1^8 and A_1^2, which directly determined the a- and b-axes of the WTe_2 flakes. After the determination of crystal structure, we fabricated the devices of WTe_2/Py (**c, d**) and WTe_2/Al_2O_3/Fe (**e, f**). This is discussed in greater detail in the "Methods" section.

c. An optical image of the WTe_2 flake before WTe_2/Py device fabrication. The yellow dashed region was exposed by EBL and then reactively etched by Cl_2.

d. The final Hall device of WTe_2/Py for spin-orbit torques. In the WTe_2/Py Hall

bar devices, current is flowing along the a- or b-axis.

e. An optical image of the WTe_2 flake before the device fabrication of $WTe_2/Al_2O_3/Fe$. The yellow dashed region was exposed by EBL and then etched by Ar.

f. The final device of $WTe_2/Al_2O_3/Fe$ for the electrical detection of spin momentum relation. In the nanoribbon devices, before the deposition of the four non-magnetic electrodes of Ti/Au, the Al_2O_3 was etched to ensure Ohmic contact between the Ti/Au and WTe_2 nanoribbons. The Fe electrode was written by the third EBL and then was deposited using e-beam evaporation. The white scale bars in **c**–**f**are10 μm.

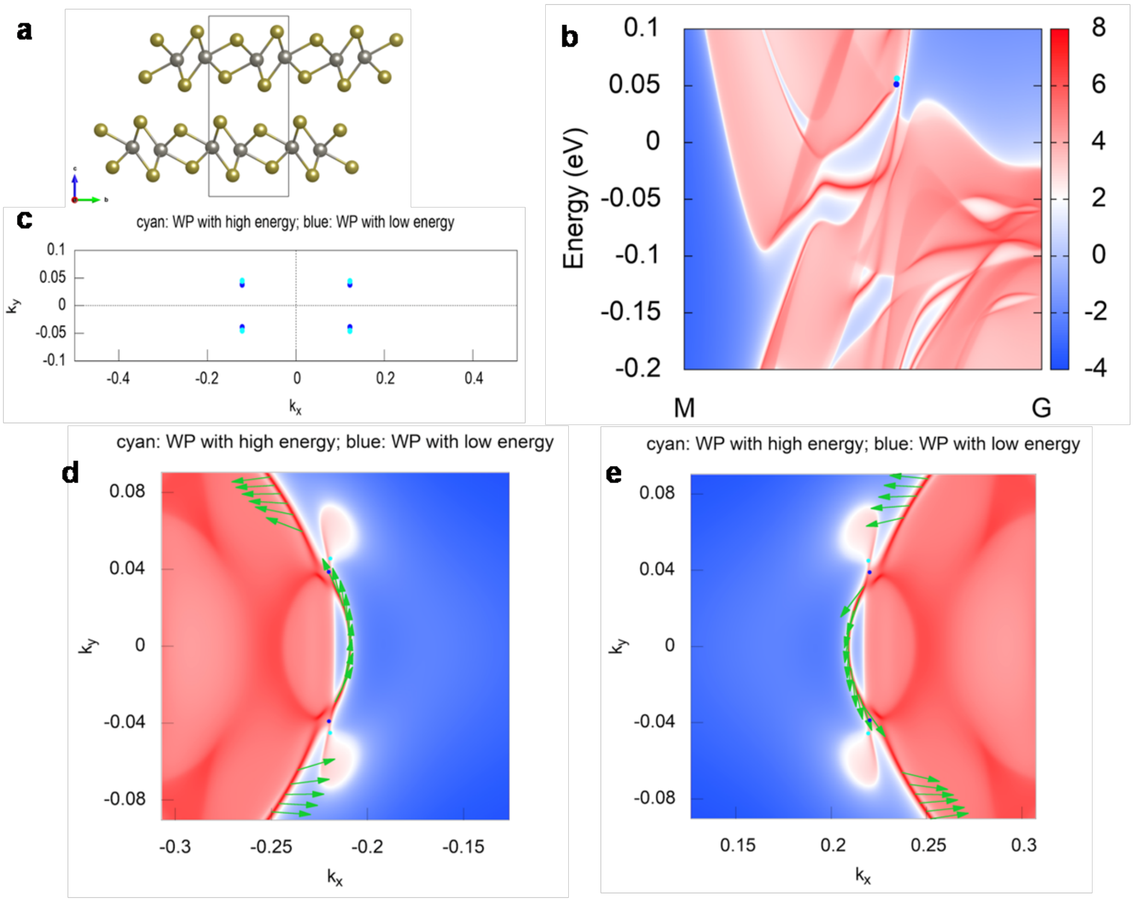

Supplementary Figure 6| Calculation of the spin-momentum locking of the surface states of WTe_2.

a. The crystal structure of 1-*T*' WTe_2.

b. The calculated band structure of WTe_2 near the Fermi level. The two dots (cyan and blue) represent the locations of the two Weyl points.

c. The location of the two Weyl points with different chiralities at the $k_z = 0$ plane.

d/e. The calculated relation between spin and momentum in surface states in different regions of the Brillouin zone. The green arrow represents the spin. To clearly illustrate the results of the following experiments, we have plotted the relation of spin and momentum in the top surface of the WTe_2. On the bottom

surface of the WTe_2, the direction of the spin was opposite to the spin in d and e. The energy between the two Weyl points (cyan point: E = 0.056 eV; blue point: E = 0.052 eV) was chosen. Based on the narrow separation between the two Weyl points with different chiralities, we know that the spin was almost tangential to the momentum of the Fermi arc. The detailed relation between the spin and momentum of the surface states and the Fermi arc in the WTe_2 are plotted in Figs. 2b–2d and Fig. 3a, respectively.

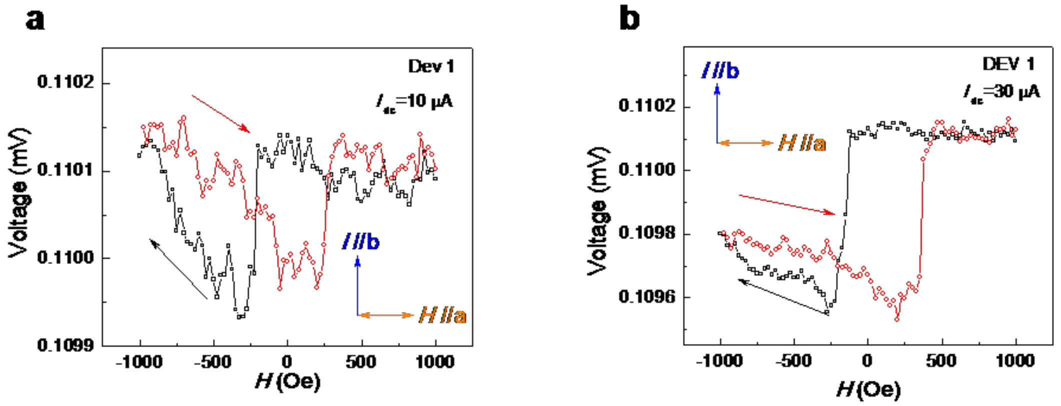

Supplementary Figure 7| Resistance hysteresis loop of the b-axis WTe_2/Al_2O_3/Fe tunnel junction.

At 2 K, the in-plane magnetic field was swept along the a-axis under the application of different DC current **a**, I_{dc} = 10 μA; **b**, I_{dc} = 30 μA. Compared to $\mathbf{I_{dc}}//a$, **H**//*b* (Fig. 2f), the hysteresis loop behavior in **a** ($\mathbf{I_{dc}}//b$, **H**//*a*) was not ideal. However, with the increase of the DC current (30 μA in **b**), the resistance switching became evident, as illustrated in Fig. 2f. The observations in **a** and **b** can be understood under the following frame. When a DC current of 10 μA is applied, the accumulated spin of the conventional surface states shown in Fig. 2d is along the a-axis, while the spin of the Fermi arc states in Fig. 3a is also accumulated, but along the b-axis. The spin of both the surface states and the Fermi arc states mix, leading to an anisotropic magnetoresistance behavior in **a,** which has been reported in topological insulators.[4] The anisotropic magnetoresistance-like switching behavior is also observed in Supplementary Fig. 11d. Nevertheless, the surface states should be more evident than the Fermi arc states due to the nearly adjacent Weyl points (Fig. 2b and Supplementary Fig. 6c). Therefore, with the increased DC current, we can observe a more typical hysteresis loop behavior in **b**, which is related to the spin generated by

the surface states in Fig. 2d.

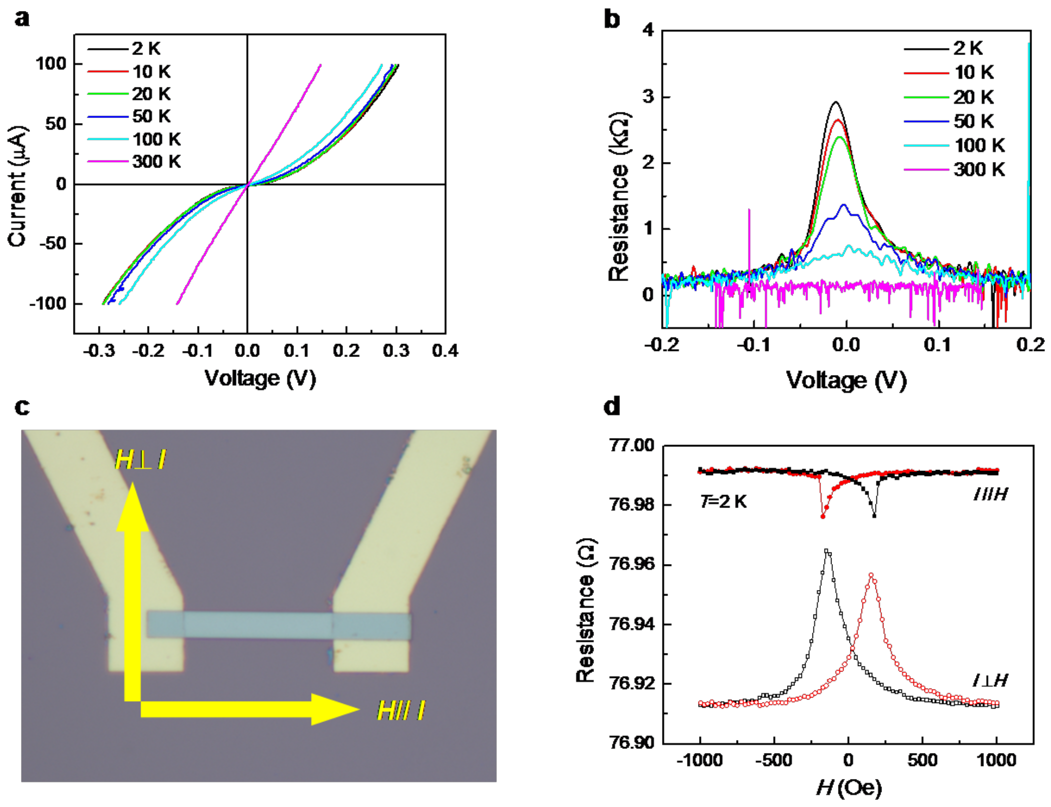

Supplementary Figure 8| Basic physical properties of the $WTe_2/Al_2O_3/Fe$ tunnel junction.

- **a.** The voltage dependence of the tunnel current of $WTe_2/Al_2O_3/Fe$ at different temperatures.
- **b.** The voltage dependence of the resistance at different temperatures.
- **c.** An optical image of the Fe (6 nm)/Ti (4 nm) device for the determination of the coercive field of ferromagnetic layer.
- **d.** The anisotropic magnetoresistance of Fe(6 nm). The coercive field for *I*//*H* and *I*⊥*H* is approximately200 Oe, which is almost the same as the switching field in the resistance hysteresis loop depicted in Figs. 2 and 3 in main text.

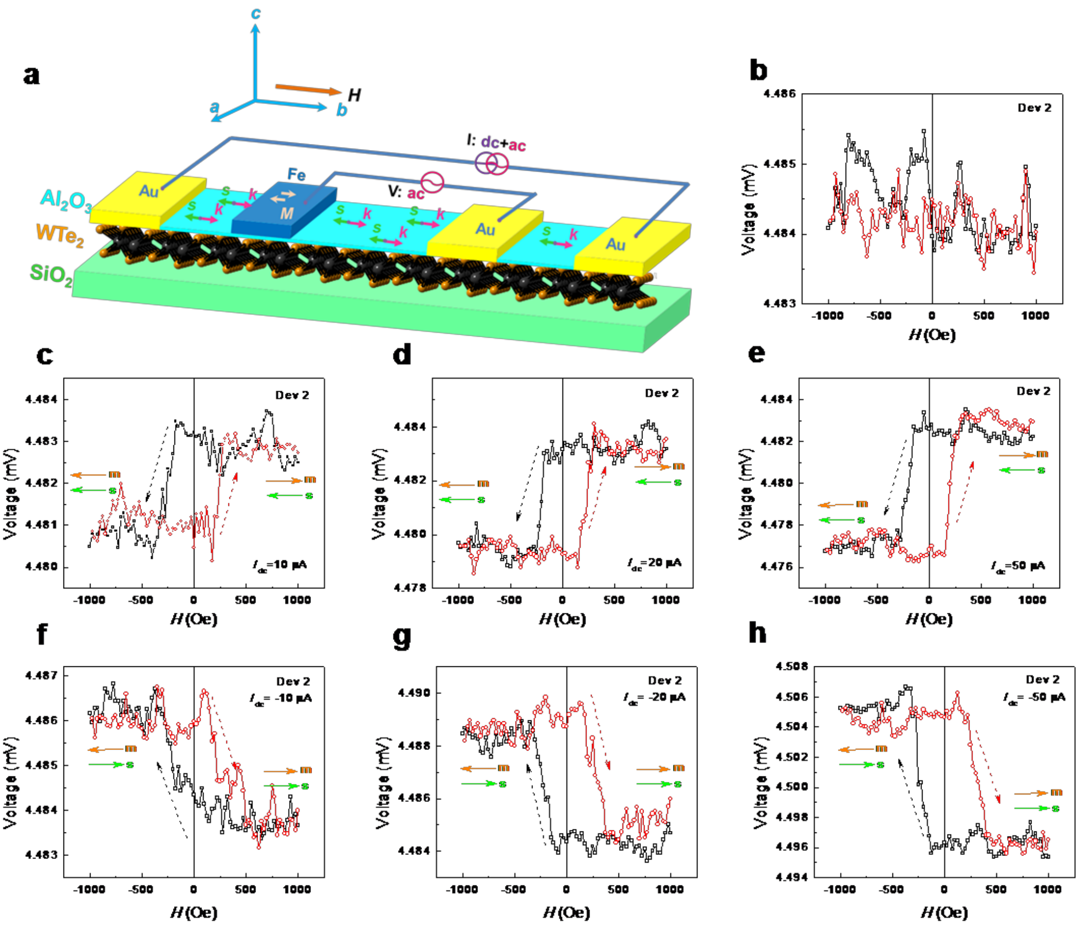

Supplementary Figure 9| Electrical detection of the spin-momentum locking of the Fermi arc inWTe_2/Al_2O_3/Fe tunnel junctions.

a. A schematic of the measurement of theWTe_2/Al_2O_3/Fe tunnel junctions (Device 2, 23 nm). To ensure the good contact of the two DC current electrodes between Au and WTe_2, we etched Al_2O_3 by Ar before the deposition of Ti/Au. During the measurement, we applied a high DC current to generate the spin accumulation in the top Fermi arc state in WTe_2. We also added a low AC current of 1 μA between two Ti/Au electrodes to detect the voltage hysteresis between Fe/Al_2O_3 and Au, using the same four-probe method as in the previous experiment.[5]

b. The field dependence of the voltage without a DC current. We did observe any

voltage hysteresis behavior without a DC current while sweeping the in-plane magnetic field. However, we did observe the voltage hysteresis loop between the Ti/Au and Fe/Al_2O_3 electrodes when a DC current was applied.

c–h. The magnetic field dependent voltage under different DC current at 2 K. The DC currents applied in the measurement of the magnetic-field-dependent voltages were 10 μA (**c**), 20 μA (**d**), 50 μA (**e**), -10 μA (**f**), -20 μA (**g**), and -50 μA (**h**), respectively. Clearly, positive and negative DC currents resulted in opposite voltage differences between the high- and low-resistance states, and these differences increased with the amplitude of the DC current. The relative orientation between the spin accumulation in the Fermi arcs and the momentum of the Fe electrodes is added in panels **c-h**. The DC-current-dependent voltage difference is summarized and plotted in Fig. 3e in main text.

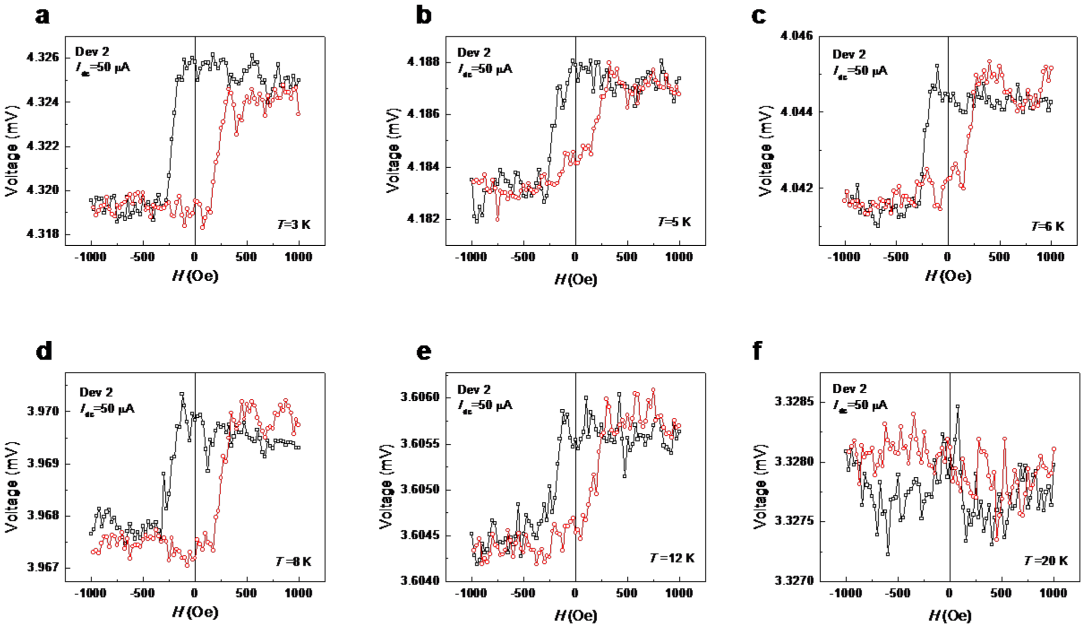

Supplementary Figure 10| Voltage hysteresis loop in $WTe_2/Al_2O_3/Fe$ tunnel junctions at different temperatures in Device 2.

As the temperature of Device 2increased from 3 K to 20 K (**a,** 3 K, **b,** 5 K, **c,** 6 K, **d,** 8 K, **e,** 12 K, and **f,** 20 K), the voltage difference between the high- and low-resistance states gradually decreased. During the measurements, a constant DC current of 50 μA was applied. The temperature-dependent voltage difference is summarized and plotted in Fig. 3f in main text.

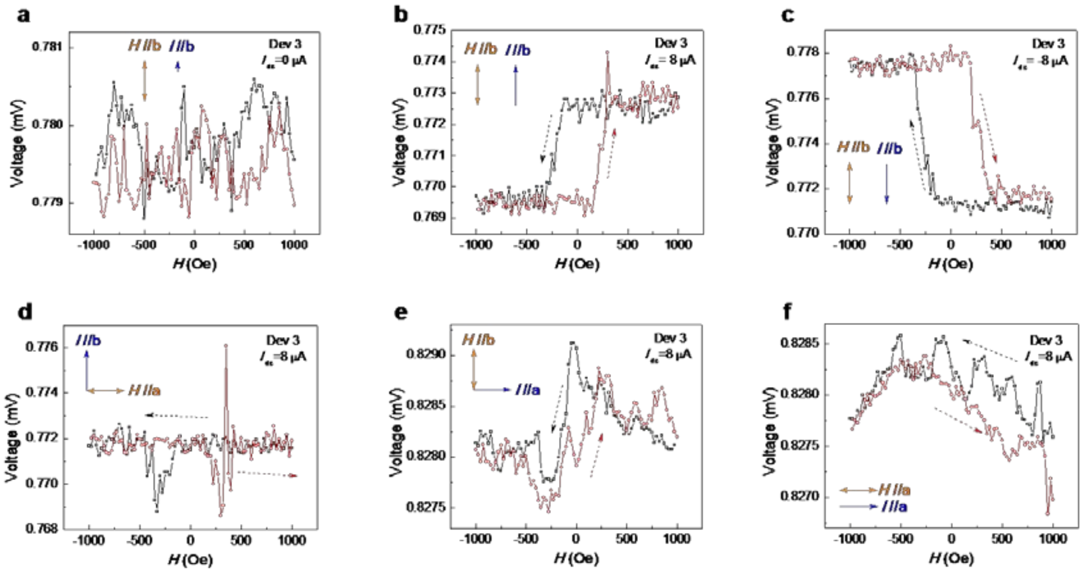

Supplementary Figure 11| Voltage hysteresis loop in $WTe_2/Al_2O_3/Fe$ tunnel junctions in Device 3.

The WTe_2 thickness of the third device is 17 nm. To reinforce our conclusion, we repeated the voltage hysteresis loop with different configurations. All of the measurements were conducted at 2 K with different configurations: **a**, **H**//b, without dc current; **b**, **H**//*b*, **I**//*b*, I_{dc} = 8 μA; **c**, **H**//*b*, **I**//*b*, I_{dc} = -8 μA; **d**, **H**//*a*, **I**//*b*, I_{dc} = 8 μA; **e**, **H**//*b*, **I**//*a*, I_{dc} = 8 μA; **f**, **H**//*a*, **I**//*a*, I_{dc} = 8 μA. All of the observations of Device 3 are consistent with those depicted in Figs. 2 and 3 in the main text and Supplementary Figures 7 and 9.

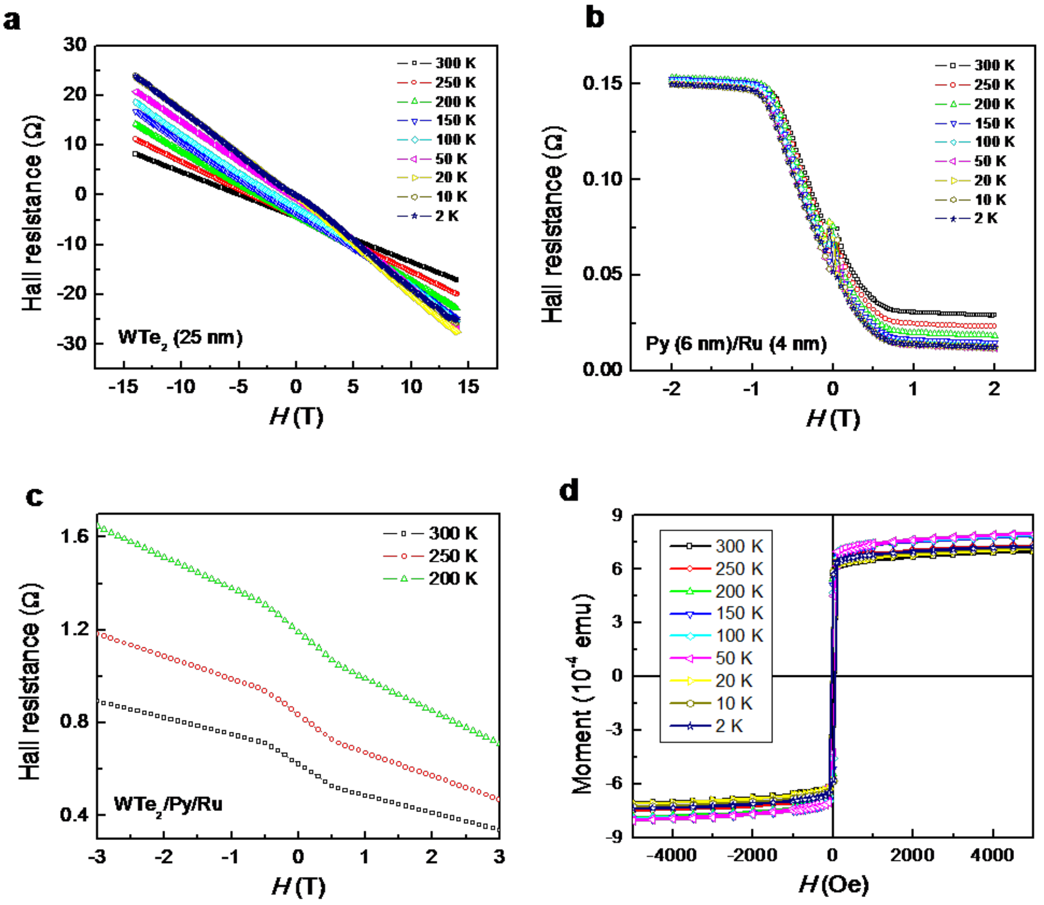

Supplementary Figure 12| Basic physical properties of WTe_2/Py bilayers.

a. The Hall effect of WTe_2 ($t \approx 25\,\text{nm}$) at different temperatures. As a semimetal with well *n*-*p* compensation,[6] the Fermi level in the bulk $WTe_2$was approximately50–60 meV below the location of the Weyl points. Typical *n*-type conduction is observed in **a**, indicating that the Fermi level is elevated and near the location of the Weyl points. We used the transport method to extract spin-orbit-torques, and the elevated Fermi level in several-layered WTe_2 enabled us to detect the effect of the Fermi arc on the spin-orbit torques.

b. The anomalous Hall effect (AHE) of the Py Hall bar ($t \approx 6\,\text{nm}$).

c. The Hall effect in the bilayer WTe_2/Py. Both the AHE of the Py and ordinary Hall effect of the WTe_2 contributed to the measured data, indicating that

current flowed along both layers.

d. The magnetic-field-dependent magnetization of Py (6 nm) at different temperatures. The magnetization of Py decreases slightly as temperature increases.

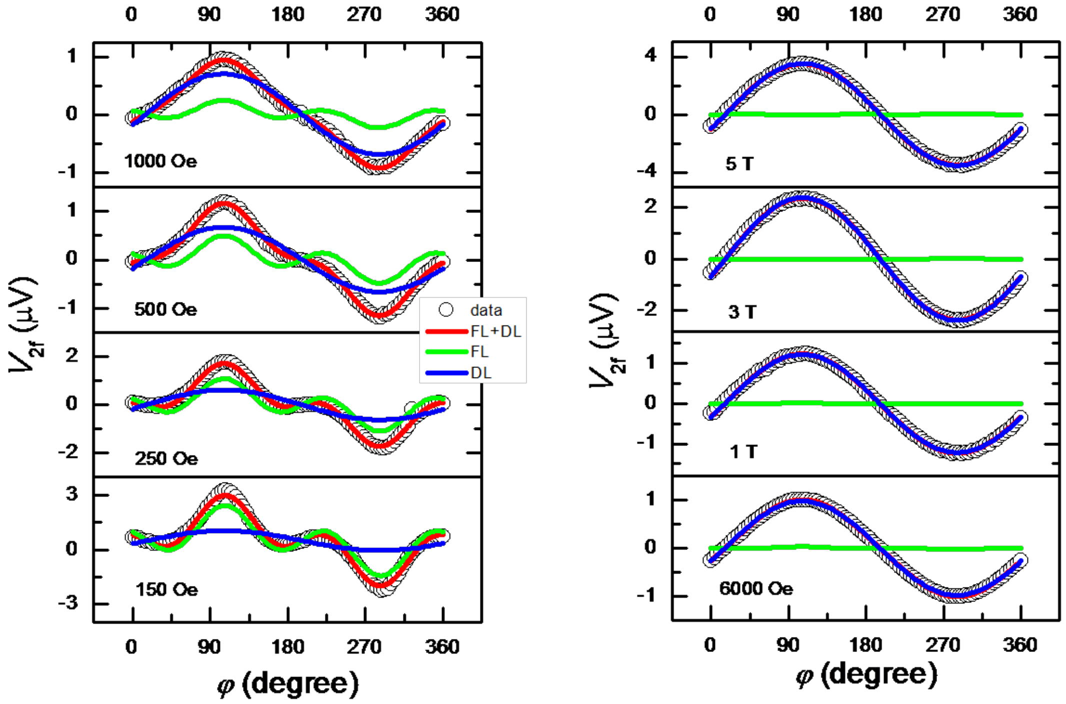

Supplementary Figure 13| Typical separation of the field-like torque and damping-like torque in the second-harmonic Hall voltage measurements

We provide the angular-dependent second-harmonic voltage of the WTe_2/Py devices under different magnetic fields and their fittings. The green and blue lines respectively represent the contribution of the field-like (FL) torque and damping-like (DL) torque of Equation 1 (as found in the main text). The red line is the sum of both contributions. Notably, the contribution of the FL torque decays rapidly as the magnetic field increases.

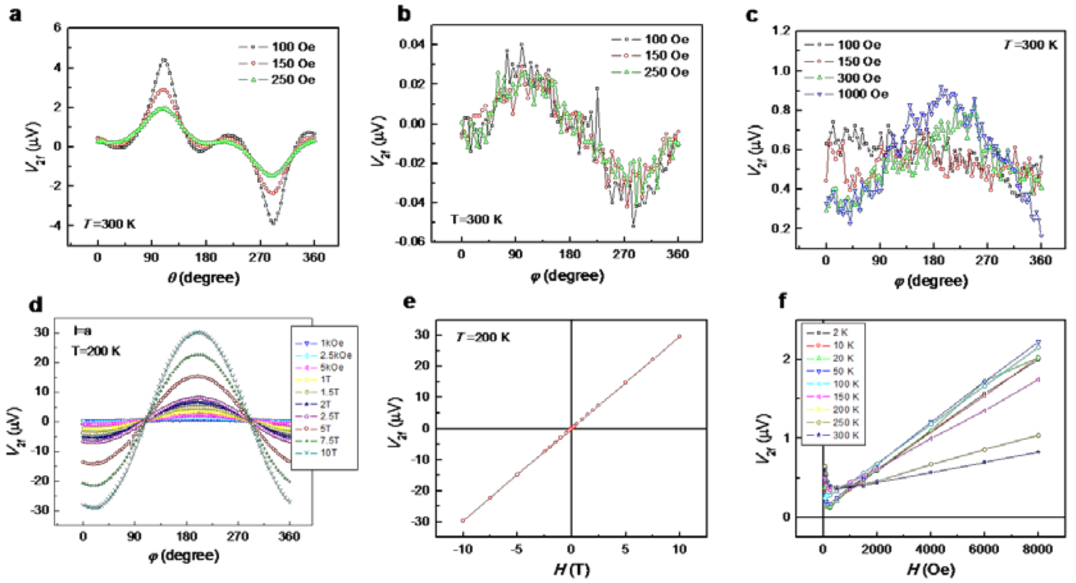

Supplementary Figure 14| Exclusion of the extrinsic effects on the second-harmonic Hall voltage.

a. The typical angular-dependent second-harmonic Hall voltage of the WTe_2 (20 nm)/Py (6 nm) devices (T = 300 K)**.**

b. The typical angular-dependent second-harmonic Hall voltage of Py (6 nm)/Ru (4 nm) for comparison.

c. The second-harmonic Hall voltage of the WTe_2 (25 nm) without the ferromagnetic Py layer under a small external magnetic field.

d. The angular-dependent second-harmonic Hall voltage of the WTe_2 (25 nm) under a large magnetic field (H>1 kOe). All the data observed in **a–d** are from devices with exactly the same dimensions (width of Hall bar 5 μm) under an AC current of 0.5 mA and a frequency of 87.8 Hz. Notably, the field-like torque originates from the interface between the WTe_2 and Py.

e. The extracted second-harmonic Hall voltage amplitude of the damping-like term ($\cos\theta$) as a function of the magnetic field of WTe_2. The data were

extracted from **d**.

f. The extracted second-harmonic Hall voltage amplitude of the damping-like term ($\cos\theta$) as a function of the magnetic field of WTe_2/Py. The strong linear background of the second-harmonic voltage in the WTe_2 is quite puzzling; since WTe_2 is nonmagnetic, it should not generate any detectable second-harmonic Hall voltage (according to Supplementary Note 1). This strong linear background of the damping-like term in the WTe_2/Py prevented us from extracting a reliable damping-like torque, spin Hall angle, and spin-current conductivity for the WTe_2. If we observe the magnetic-field-dependent damping-like term at different temperatures in **f**, we can easily find both the contributions of the ANE and the rather puzzling linear background. The strong linear background probably comes from the chiral anomaly induced planar Hall effect of WTe_2.[7-9]

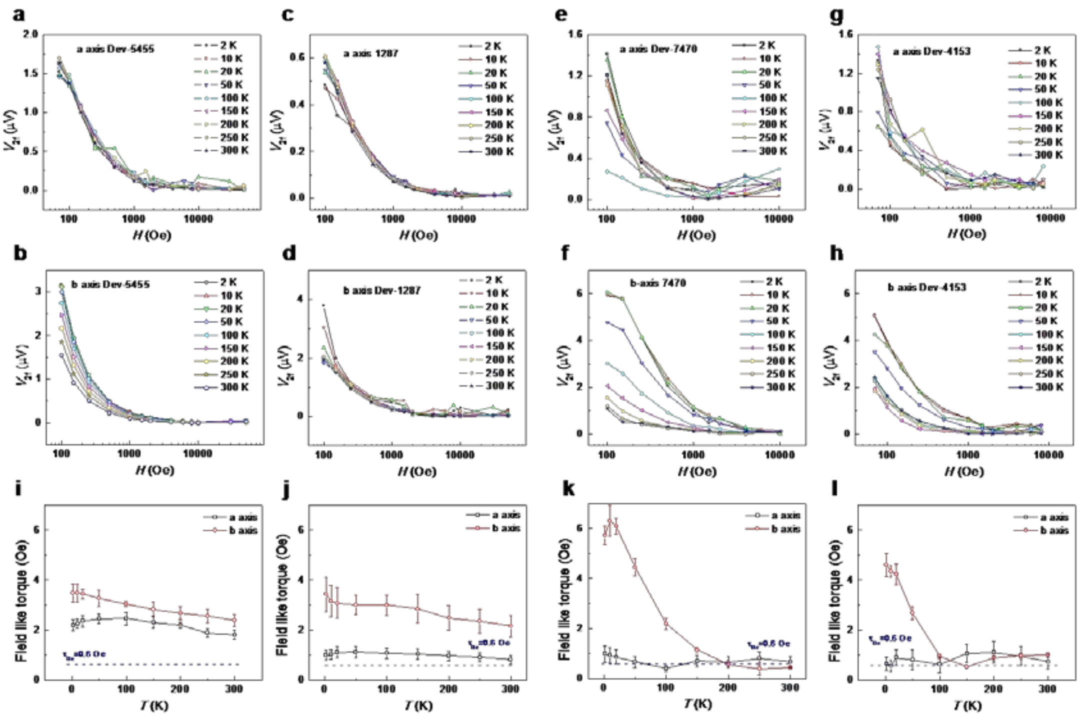

Supplementary Figure 15| Anisotropic magnetic-field-dependent field-like torque term in all four WTe_2/Py devices.

The WTe_2 thicknesses in all four devices are 5.6 nm (**a, b, i**), 7.0 nm (**c, d, j**), 20.0 nm (**e, f, k**), and 31.0 nm (**g, h, l**), respectively. All of the field-dependent second-harmonic Hall voltages of the FL term (in Fig. 5 of the main text) are shown in **a–l.** We can easily obtain the FL torque at each temperature through fitting the individual curves into Equation 1(as demonstrated in the main text). The temperature dependence of the fitted FL torque along the different directions is given in **i–l**. The calculated Oersted field was also added into **i–l** for comparison. The panels **a, c, e,** and **g** contain field dependent second harmonic Hall voltages related to FL torques with the current along the a-axis of WTe_2, whereas the panels **b, d, f,** and **h** give field dependent second harmonic Hall voltages related to FL torques with the current along the b-axis of WTe_2. The error bar comes from the combination of the 20% standard

error and fitting processes.

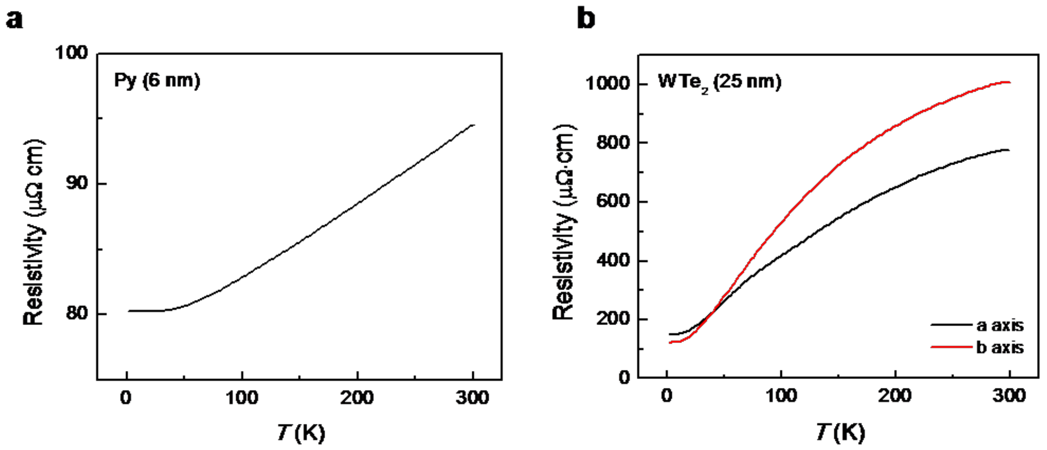

Supplementary Figure 16| Temperature dependence of the resistivity of Py and WTe_2 nanoribbons.

The metallic behavior was observed in both the Py (**a**) and WTe_2 (**b**). The thicknesses of the Py and WTe_2 were 6 nm and 25 nm, respectively. The resistivity of the WTe_2 was anisotropic. The resistivities of the WTe_2 along the a- and b-axes at 2 K were 148 μΩ cm and 121 μΩ cm, respectively. Therefore, the current fraction in the WTe_2 was slightly different (less than 8%) in both the a- and b-axes of the WTe_2/Py under a total current of 0.5 mA. However, this anisotropic current fraction in the WTe_2 cannot lead to such high anisotropic behavior in the FL torque at low temperatures. In particular, the Oersted field generated by the current is further excluded in Supplementary Fig. 17.

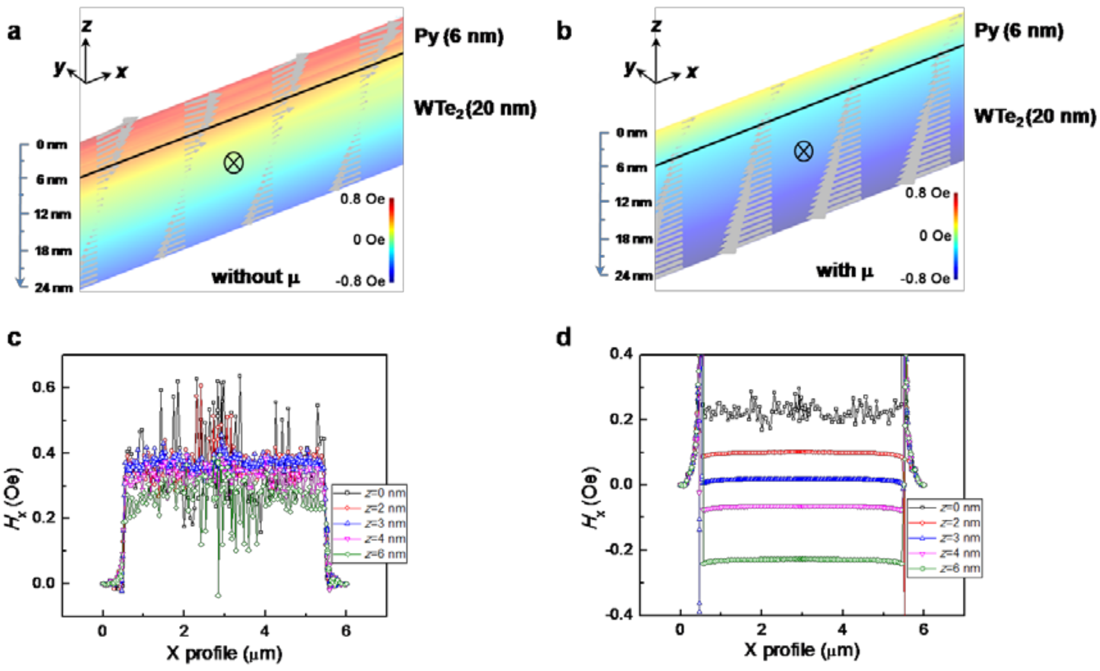

Supplementary Figure 17| Simulation of the Oersted field in WTe_2/Py bilayers.

The current (0.5 mA) was applied along the *y*-axis of the WTe_2 (20 nm)/Py (6 nm) bilayers. The distribution of the current fraction in the WTe_2 and Py was calculated according to their resistivities in Supplementary Fig. 16. We used the COMSOL Multiphysics software to conduct the simulation. We estimated the Oersted field generated by the current without (**a**) and with (**b**) considering the magnetic permeability of Py. The direction and strength of the Oersted field the in *xz* plane are plotted in **a** and **b**. If we had not considered the permeability of Py, the Oersted field in the Py would have been along the positive direction of *x*-axis and thus would have been mainly generated by the current in the WTe_2 (**a**). As plotted in **c,** the Oersted field is observed at different positions in Py. The value in **c** is almost 0.3–0.4 Oe and lower than the value that was estimated using Ampere's law (~0.62 Oe). If the permeability of Py is taken into account, the direction of the Oersted field in Py (*z* range: 3–6 nm) is, surprisingly, reversed. As plotted in **d**, the Oersted field is observed

at different positions in Py. The negative Oersted field in the *z* region, from 3 to 6 nm, should be ascribed to the current contribution of the upper region of Py (*z* range: 0–3 nm) and the strong permeability of Py. The average Oersted field in **b** and **d** should be close to zero. Nevertheless, the average Oersted field generated by the current in both cases should be smaller than the result of the calculation (~0.62 Oe) that used Ampere's law. This simulation and estimation further demonstrate the exclusion of the Oersted field.

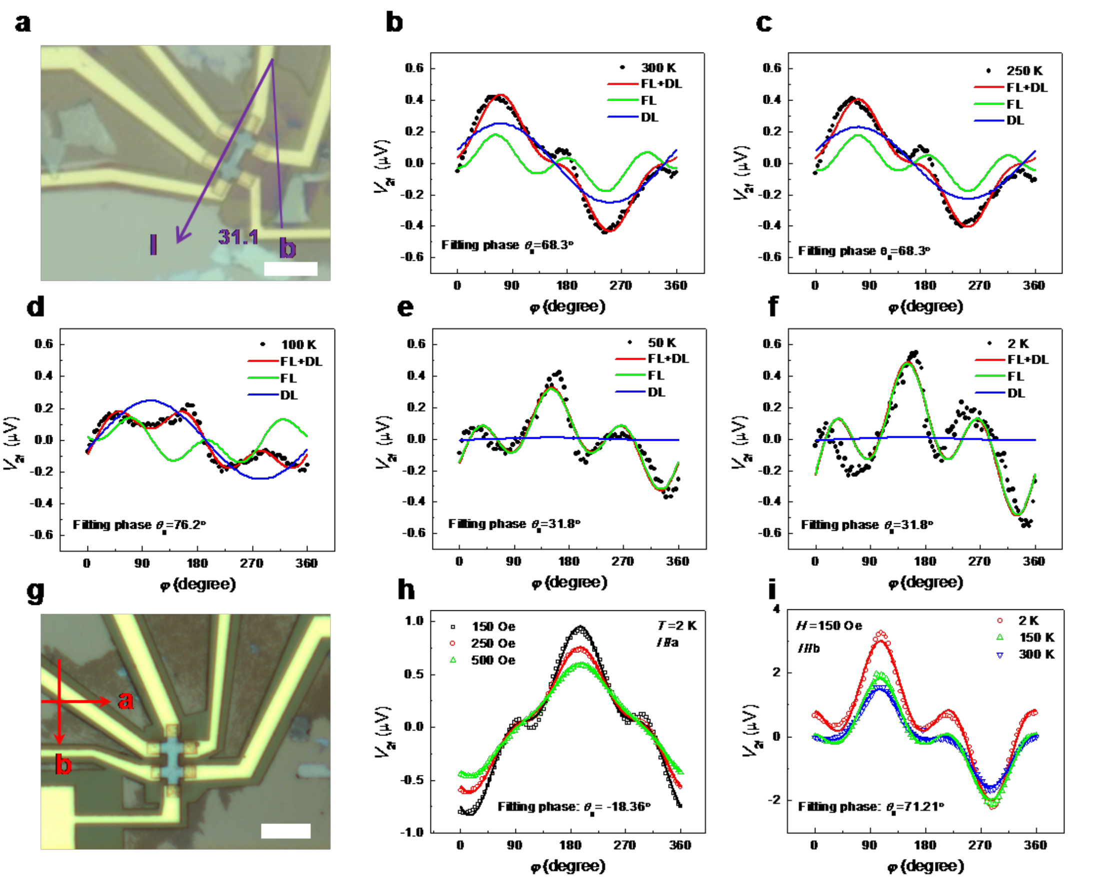

Supplementary Figure 18| Phase shift in the second-harmonic Hall voltage of the WTe_2/Py.

We fabricated a Hall bar device with the current direction approximately 31° degrees away from the b-axis of the WTe_2, as indicated in the optical image of **a**. As the temperature decreased below 100 K, a clear phase shift of the FL torque, depicted in **b–f**, was observed. The fitting phases θ_0 by Eq. (1) (as found in the main text) are 68.3° (**b, c**) and 31.8° (**e, f**), respectively. For comparison, we also give the angular-dependent second-harmonic Hall voltage with the current along the a- and b-axes. The optical image of the device is given in **g**. Using Eq.(1) in main text again, we found that the fitting phases θ_0 with the current along the a- and b-axes WTe_2/Py

(**h** and **i**) were-18.4° and 71.2°,respectively, and the fitting phase difference in **h** and **i** is almost exactly 90°.

The fitting phase θ_0 of the b-axis WTe_2/Py device with the current along the b-axis is 71.2°, which is almost the same as the fitting phase θ_0 of the device in **a** (68.3°) at high temperatures. However, the shift of the fitting phase θ_0 in this device (**a)** from high to low temperatures is approximately 36° (68.3°–31.8°≈36°), which is quite close to the angle of approximately 31° between the b-axis and the current direction of the Hall device. Because the spin in the Fermi arc (T<100 K) is locked tangential to the momentum along the b-axis (Fig. 3a), only the component of the current along the b-axis in the Hall device (**a**) accumulates a spin. Hence the accumulated spin will generate a stronger spin-orbit torque in the ferromagnetic layers with the phase shift at low temperatures. For comparison, no phase shift was observed in the device with the current exactly along the b-axis (**g** and **i)** when the temperature increased from 2 K to 300 K. This is strong evidence that the anisotropic spin-orbit torque at low temperatures in WTe_2/Py is closely related to the spin accumulated in the topological Fermi arc states.

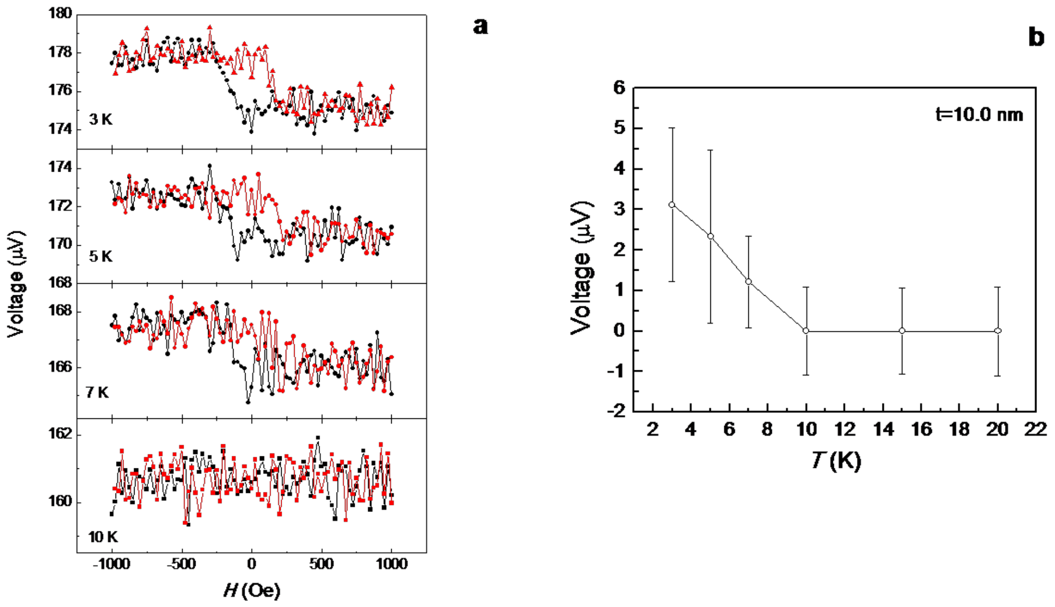

Supplementary Figure 19| Spin momentum locking detection in the device with a WTe_2 thickness of 10.0 nm. a The voltage as a function of in-plane magnetic field at different temperatures. **b** The temperature dependent voltage difference. The critical temperature that hysteresis behavior vanishes is near 7-10 K. During the measurements, a DC current of 20 μA and an AC current of 1 μA are applied to measure the tunnel resistance.

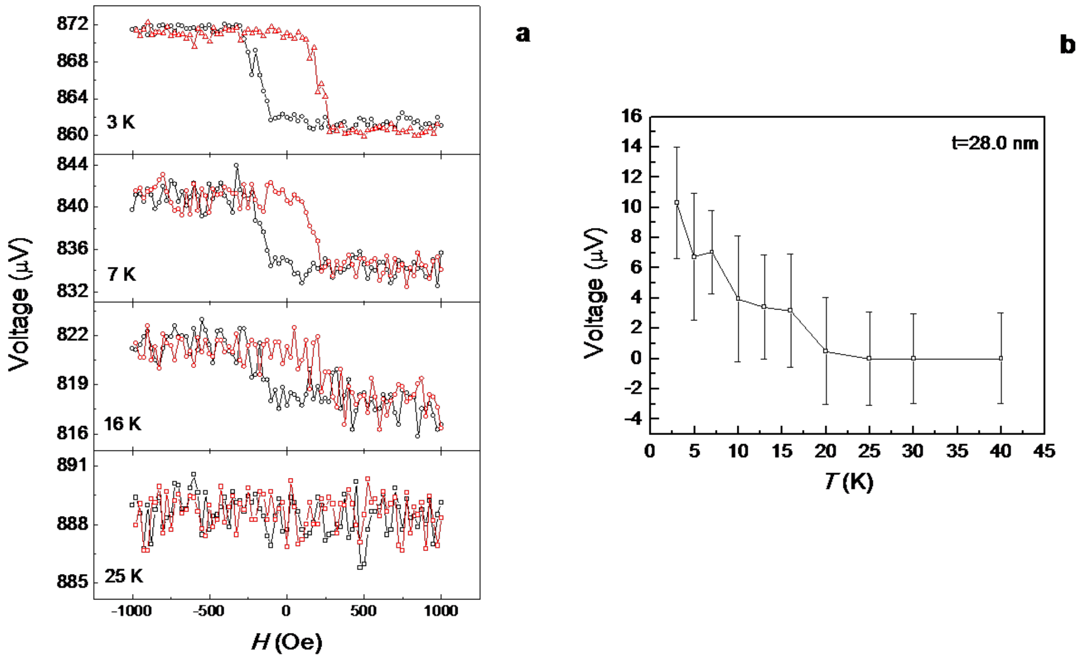

Supplementary Figure 20| Spin momentum locking detection in the device with a WTe_2 thickness of 28.0 nm. a The voltage as a function of in-plane magnetic field at different temperatures. **b** The temperature dependent voltage difference. The critical temperature that hysteresis behavior vanishes is near 15-20 K. During the measurements, a DC current of 20 μA and an AC current of 1 μA were applied to measure the tunnel resistance.

Supplementary Note 1

Derivative of spin-orbit torques through a second-harmonic model

The second-harmonic method considers the anisotropy and Zeeman energy, and the total energy is

$$E = -K_{out}\cos^2\theta - K_{in}\sin^2\varphi\sin^2\theta - \boldsymbol{M}\bullet\boldsymbol{H}\ , \tag{1}$$

where K_{out} and K_{in} respectively represent the effective out-of-plane and in-plane anisotropy energy; θ and φ respectively represent the polar and azimuthal angles of the magnetization moment $\mathbf{M}$. This moment is defined as

$$\boldsymbol{M} = M_S\boldsymbol{m} = M_S\left(\cos\varphi_m\sin\theta_m, \sin\varphi_m\sin\theta_m, \cos\theta_m\right). \tag{2}$$

M_s represents the saturated magnetization, and $\boldsymbol{m}$ represents the unit vector of the moment. The external field $\boldsymbol{H}$ is defined as

$$\boldsymbol{H} = H\left(\cos\varphi_H\sin\theta_H, \sin\varphi_H\sin\theta_H, \cos\theta_H\right). \tag{3}$$

By solving the equations $\frac{\partial E}{\partial\theta} = 0, \frac{\partial E}{\partial\varphi} = 0$ to calculate the minimum energy, we obtain the equilibrium value of the magnetization angle $\left(\theta_M, \varphi_M\right)$.

After we apply the current, the current-induced field ΔH, including both the Oersted field and the effective field caused by the spin-orbit torques, slightly alters the magnetization with a small modulation angle $\left(\Delta\theta, \Delta\varphi\right)$.

If we assume that the external field is in-plane $\left(\theta_H = \pi/2\right)$ and that the in-plane anisotropy is much smaller than the external field $\left(\left|H_A\right| \ll \left|H\sin\theta_H\right|\right)$, the modulation angle can be simplified as follows:

$$\Delta\theta = \frac{\Delta H_Z}{H_K - H}$$
$$\Delta\varphi = \frac{-\Delta H_X \sin\varphi_H + \Delta H_Y \cos\varphi_H}{H - H_A} . \quad (4)$$

The relation between the Hall resistance and the modulation angle can be established by considering the anomalous Hall effect and planar Hall effect. Based on previous reports, the Hall resistance can be expressed as

$$R_H = R_A \cos\theta + R_P \sin^2\theta \sin 2\varphi , \quad (5)$$

where R_A and R_P represent the coefficients of the AHE and PHE. If the current-induced field is small enough in comparison to the external field, the modulation angle $\Delta\theta, \Delta\varphi$ should be slight. Thus, the equation above can be simplified as

$$R_H = R_P \sin 2\varphi_m + \left(\Delta\varphi \cdot 2R_p \cos 2\varphi_m - \Delta\theta \cdot R_A\right). \quad (6)$$

When an alternating current $\left(i = I \sin\omega t\right)$ is applied, the current-induced field also oscillates at the same frequency as the current. Hence, we can use $\Delta\theta \sin\omega t, \Delta\varphi \sin\omega t$ to replace $\Delta\theta, \Delta\varphi$. Thus, the Hall voltage could be expressed as

$$V_H = \left[R_P \sin 2\varphi_m \sin\omega t + \left(\Delta\varphi \cdot 2R_p \cos 2\varphi_m - \Delta\theta \cdot R_A\right)\sin^2\omega t\right] I . \quad (7)$$

Then, the Hall voltage can be separated into three parts determined by frequency:

$$V_H = V_0 + V_\omega \sin\omega t + V_{2\omega} \cos 2\omega t . \quad (8)$$

Since the zero-order term can be easily influenced by the DC offset of the sinusoidal current, the first- and second-harmonic terms become more useful to evaluate the modulation angle:

$$V_{\omega} = R_P \sin 2\varphi_m \cdot I$$
$$V_{2\omega} = \left(-\Delta\varphi \cdot R_p \cos 2\varphi_m + \frac{1}{2}\Delta\theta \cdot R_A \right) I \,' \tag{9}$$

Considering Eq. (4) and $\varphi \equiv \varphi_m = \varphi_H$, we have

$$V_{\omega} = R_P \sin 2\varphi \cdot I$$
$$V_{2\omega} = \left(\frac{\Delta H_X \sin\varphi - \Delta H_Y \cos\varphi}{H - H_A} \cdot R_p \cos 2\varphi + \frac{1}{2} \frac{\Delta H_Z}{H_K - H} \cdot R_A \right) I \,. \tag{10}$$

To find the FL and damping-like torques quantitatively, we need to introduce the Landau-Lifshitz-Gilbert equation:

$$\frac{d\boldsymbol{m}}{dt} = -\gamma \boldsymbol{m} \times \left[\boldsymbol{H} + \alpha \left(\boldsymbol{m} \times \boldsymbol{H} \right) + H_{FL}\boldsymbol{\sigma} + H_{AD}\left(\boldsymbol{m} \times \boldsymbol{\sigma} \right) \right], \tag{11}$$

where γ represents the gyromagnetic ratio, α represents the Gilbert damping coefficient, $\boldsymbol{H}$ represents the external field, and $\boldsymbol{\sigma}$ represents the normalized spin direction among electrons absorbed by the ferromagnetic layer. Moreover, $\boldsymbol{H_{FL}} = H_{FL}\boldsymbol{\sigma}$ and $\boldsymbol{H_{AD}} = H_{AD}\left(\boldsymbol{m} \times \boldsymbol{\sigma} \right)$ represent the effective field induced by the FL and DL torques, respectively. When rotating the field in-plane, we have $\boldsymbol{m} = \left(\cos\varphi, \sin\varphi, 0 \right)$ and $\boldsymbol{\sigma} = \left(0,1,0 \right)$, which leads to $\boldsymbol{H_{FL}} = \left(0, H_{FL}, 0 \right)$ and $\boldsymbol{H_{AD}} = \left(0, 0, H_{AD} \cos\varphi \right)$. By developing this into Eq.(10), we have

$$V_{2\omega} = \left(\frac{-H_{FL} \cos\varphi}{H - H_A} \cdot R_p \cos 2\varphi + \frac{1}{2} \frac{H_{AD} \cos\varphi}{H_K - H} \cdot R_A \right) I \tag{12}$$

Supplementary Note 2

As we observed in Fig. 3f and Supplementary Figures 19-20, the critical temperatures that hysteresis behavior vanishes are 7-10 K, 12-15 K and 15-20 K for

10.0 nm, 23.0 nm and 28.0 nm thick samples. The critical temperature does not seem to agree well with the observation in spin-orbit-torques in Figures 4-5 in main text. There are two reasons that account for the difference in both experiments.

First of all, we have to point out that the interfaces in the samples used to measure two effects are different, i.e. two interfaces in the sample of ferromagnet-insulator-Weyl used in spin momentum locking measurements, and one interface in the sample of ferromagnet-Weyl used in spin-orbit torques measurements. The effective spin polarization detected by ferromagnet and ferromagnet/insulator should be different.[10] Only when the thickness of the tunnel barrier is thick enough, the spin polarization by ferromagnet/insulator will approach the bulk spin polarization of ferromagnet.[10] Normally, the effective spin polarization in ferromagnet/insulator is smaller than that in bulk ferromagnet. Therefore, the critical temperature obtained in spin momentum locking experiment using a device of WTe_2/Al_2O_3 should be smaller than that obtained from the spin orbit torque measurement using a WTe_2/Py device for the thick tunneling barrier lay of Al_2O_3(3 nm).

Second, during the device fabrication process, there are two more E-beam lithography (EBL) steps in making the devices used in spin momentum locking detection than in making the devices used in spin orbit torques measurements. Particularly, the WTe_2/Al_2O_3 layer has to be exposed to the e-beam lithography photoresist (PMMA). The quality of WTe_2 crystal might be deteriorated by the fabrication process, which might consequently damage the robust of topological Fermi arc states. Therefore, in comparison the critical temperature observed in the

spin orbit torques measurements, the decrease of critical temperature observe in the measurements of spin momentum locking should be expected.

Most importantly, with the increase of WTe_2 thickness, the critical temperature in spin momentum locking detection increases from 7-10 K (10.0 nm) to 15-20 K (28.0 nm). This tendency agrees well with the observation in spin orbit torques measurements (Figure 5 in main text).

Supplementary Note 3

Surface conduction and bulk conduction in WTe_2

From the data of Hall effect of WTe_2flake in Supplementary Figure 12a, we know the dominated bulk carrier in WTe_2 flakes is *n*-type with a carrier density of $1.41(\pm0.02)\times10^{20}$ cm^{-3}. This carrier density in our WTe_2 flakes is almost twice that of the *n*-type carrier density of bulk WTe_2 reported previously.[6] The higher bulk carrier density of electrons in our very thin plates of several tens nanometers can be understood as following. In the large, single crystalline WTe_2bulk, both low density of electrons and holes contribute nearly equally to the electrical conduction for its semimetal characteristics.[11]. With decreasing the thickness of WTe_2 to the nm level, the Fermi level will shift upward near the Weyl point.[12] Consequently the dominated carrier changes to *n*-type and much more electrons (could be doubled) will be involved into the electrical transport.

To estimate the surface carrier density, we need to calculate from the Weyl orbit

quantum oscillation in Figure 1 shown in main text, which is consist of two topological Fermi arcs and two bulk chiral Landau levels. According to Figure 1b, the frequency of Weyl orbit is ~82.0 T, we can obtain the carrier density of 2D surface states $n_{\mathrm{SS}} = ef_{\mathrm{SdH}} / h = 1.90\times10^{12}\ \mathrm{cm}^{-2}$.[5,13]

According to our previous quantum oscillation analysis,[12] the Fermi velocity is $v_{\mathrm{F}} = 3.1\times10^{5}\ \mathrm{m/s}$, quantum scattering time is $\overline{\tau} = 1.6\times10^{-13}\ \mathrm{s}$, the length of Fermi arc is $k_{\mathrm{F}} = 0.032\ \mathrm{A}^{-1}$.[12] Therefore, the quantum mobility of 2D Fermi arc state can be calculated as $\mu = ev_{\mathrm{F}}\overline{\tau} / \hbar k_{\mathrm{F}} = 2520\ \mathrm{cm}^{2}/\mathrm{V}\cdot\mathrm{s}$. Thus, the surface state conductance is $\sigma = en_{\mathrm{2D}}\mu_{\mathrm{2D}} = 0.766\ \mathrm{mS}$, while the total conductance of WTe_2 at 2 K is $\sigma_{\mathrm{total}} = 1/R_{\mathrm{sheet}} = 42\ \mathrm{mS}$. The ratio of surface conduction to total conduction is, therefore, $\alpha = \sigma_{\mathrm{2D}} / \sigma_{\mathrm{total}} = 1.8\%$, This rather low proportion of surface state conduction is reasonable, because topological Fermi arc states and high density bulk carrier coexist near the Fermi level ($n_{\mathrm{SS}} = 1.90\times10^{12}\ \mathrm{cm}^{-2}$, and $n_{\mathrm{bulk}} = 1.41\times10^{20}\ \mathrm{cm}^{-3}$). This case is opposite to the relative large surface state conduction in topological insulators.[5] Nevertheless, the 2D surface conduction in WTe_2/Py can be obviously enhanced due to the formation of Rashba interface, as shown in Figures 1c and 1d in main text.

Supplementary References

1 Jiang, Y., Gao, J. & Wang, L. Raman fingerprint for semi-metal WTe_2 evolving from bulk to monolayer. *Sci. Rep.-Uk* **6**, 19624 (2016).

2 Kim, M. *et al.* Determination of the thickness and orientation of few-layer tungsten ditelluride using polarized Raman spectroscopy. *2D Materials* **3**, 034004 (2016).

3 Song, Q. *et al.* The in-plane anisotropy of WTe_2 investigated by angle-dependent and polarized Raman spectroscopy. *Sci. Rep.-Uk* **6**, 29254 (2016).

4 Li, C. *et al.* Electrical detection of charge-current-induced spin polarization due to spin-momentum locking in Bi_2Se_3. *Nat. Nanotechnol.* **9**, 218-224 (2014).

5 Tang, J. *et al.* Electrical detection of spin-polarized surface states conduction in $(Bi_{0.53}Sb_{0.47})_2Te_3$ topological insulator. *Nano Lett.* **14**, 5423-5429 (2014).

6 Zhu, Z. *et al.* Quantum oscillations, thermoelectric coefficients, and the fermi surface of semimetallic WTe_2. *Phys. Rev. Lett.* **114**, 176601 (2015).

7 Burkov, A. Giant planar Hall effect in topological metals. *Phys. Rev. B* **96**, 041110 (2017).

8 Nandy, S., Sharma, G., Taraphder, A. & Tewari, S. Chiral anomaly as the origin of the planar Hall effect in Weyl semimetals. *Phys. Rev. Lett.* **119**, 176804 (2017).

9 Wang, Y. *et al.* Planar Hall effect in type-II Weyl semimetal WTe_2. Preprint at https://arxiv.org/abs/1801.05929 (2018).

10 Slonczewski, J. C. Conductance and exchange coupling of two ferromagnets separated by a tunneling barrier. *Phys. Rev. B* **39**, 6995-7002 (1989).

11 Ali, M. N. *et al.* Large, non-saturating magnetoresistance in WTe_2. *Nature* **514**, 205 (2014).

12 Li, P. *et al.* Evidence for topological type-II Weyl semimetal WTe_2. *Nat. Commun.* **8**, 2150 (2017).

13 Mallinson, R., Rayne, J. & Ure Jr, R. de haas-van alphen effect in *n*-type Bi_2Te_3. *Phys. Rev.* **175**, 1049-1056 (1968).
